# The burden of mental disorders in Asian countries, 1990–2019: an analysis for the global burden of disease study 2019

**DOI:** 10.1038/s41398-024-02864-5

**Published:** 2024-03-28

**Authors:** Qi Chen, Shu Huang, Huan Xu, Jieyu Peng, Ping Wang, Shiqi Li, Jinxi Zhao, Xiaomin Shi, Wei Zhang, Lei Shi, Yan Peng, Xiaowei Tang

**Affiliations:** 1 Department of Gastroenterology, the Affiliated Hospital of Southwest Medical University, Luzhou, China; 2 Nuclear Medicine and Molecular Imaging Key Laboratory of Sichuan Province, Luzhou, China; 3 Department of Gastroenterology, Lianshui County People’ Hospital, Huaian, China; 4 Department of Gastroenterology, Lianshui People’ Hospital of Kangda College Affiliated to Nanjing Medical University, Huaian, China

**Keywords:** Depression, Schizophrenia

## Abstract

Mental disorders are the leading contributors to the globally nonfatal burden of disease. This study was aimed to estimate the burden of mental disorders in Asian countries. Based on GBD 2019, the prevalence and disability-adjusted life of years (DALYs) rates with 95% uncertainty intervals (UI) were estimated in Asian countries. Predictions for the future burden of 8 selected countries, ranks of the burden of mental disorders and correlations with Sociodemographic Index (SDI) were also estimated. During the past 3 decades, while the number of DALYs of mental disorders increased from 43.9 million (95% UI: 32.5–57.2) to 69.0 million (95% UI: 51.0–89.7), the age-standardized rates of DALYs of mental disorders remained largely consistent from 1452.2 (95% UI: 1080.16–1888.53) per 100,000 population in 1990 to 1434.82 (95% UI: 1065.02–1867.27) per 100,000 population in 2019, ranked as the eighth most significant disease burden in Asia in 2019. Depressive disorders (37.2%) were the leading contributors to the age-standardized DALY rates of mental disorders in Asia, followed by anxiety disorders (21.5%). The age-standardized DALY rates in females were higher than their male counterparts, both peaked at 30-34 years. The age-standardized DALY rates were predicted to remain stable, with the number of DALYs presented an upward trend in the future. There was no significant correlation between the burden of mental disorders and SDI. All mental disorders ranked higher in 2019, compared in 1990. To reduce this burden, urgent measures for prevention, treatment, and rehabilitation for mental disorders need to be taken by Asian governments.

## Introduction

Mental illness is growingly recognized as the leading contributor to the burden of disease, although this burden largely manifested in disability rather than mortality [1, 2]. The findings from the 2009 WHO World Mental Health study indicated that mental disorders might frequently occur either with or without any comorbidity, and their symptoms frequently become apparent at a young age, resulting in considerable adverse socioeconomic impact [3]. However, mental disorders were historically not a global health priority, with services for them typically been neglected [4]. Emphasized by the Lancet Commission on global mental health and sustainable development, mental health, a fundamental human right, is crucial for the development of all countries [5]. Scaling up services for individuals impacted by mental disorders and ensuring their access to care and dignity as fundamental human rights are of great importance.

The Global Burden of Disease Study 2019 (GBD 2019), as a comprehensive international effort, including the measurement of the mental disorders burden, could be exploited to update the investigations during the period 1990–2019 on mental disorders data, which may vary greatly due to the rapid progress in diagnostic instrument technology and medical intervention strategies.

Although existing research, including studies on the global burden of mental disorders and of specific subtypes (like schizophrenia), mentioned Asia, there were no in-depth analyses of the burden of mental disorders focused in Asia by sex, age, year, and country [1, 6, 7]. To fill the gap, in this study, we aimed to estimate the mental disorders burden in Asia by sex, age group, subtypes of mental disorders and countries between 1990 to 2019, based on GBD 2019. Furthermore, we performed predictions of the disease burden resulting from mental disorders and analyses of the association between the mental disorders burden and the sociodemographic index (SDI).

## Methods

### Case definition and categorization

Mental disorders, categorized into 10 major groups among the Level 3 causes of the Global Burden of Disease (GBD) 2019, include depressive disorders, anxiety disorders, schizophrenia, bipolar disorder, conduct disorder, autism spectrum disorders, eating disorders, idiopathic developmental intellectual disability, attention-deficit hyperactivity disorder and other mental disorders. In the GBD 2019, cases were selected according to the International Classification of Disease (ICD) criteria and the Diagnostic and Statistical Manual of Mental Disorder (DSM), with various editions of ICD (both ICD-9 and ICD-10) and DSM (including DSM-III, DSM-IIIR, DSM-IV, DSM-IV-TR, and DSM-V) utilized in the course of this research [8, 9].

### Data sources

The GBD 2019 was formulated by GBD collaborators to offer a comprehensive assessment of the burden for 369 diseases and injuries over the past 3 decades, which covers 204 countries and regions. Data were sourced from the Global Health Data Exchange query tool (VizHub - GBD Results (healthdata.org)). The data from GBD 2019 resources enabled us to inquire about factors including all risks, causes, impairments, and injuries, categorized by their nature, measurements of deaths, the disability-adjusted life of years (DALYs), years lived with disability (YLDs), years of life lost (YLLs), incidence, prevalence, and so on from 1990 to 2019, with metrics of rate, percent and number stratified by sex, age, regions and territories. Population predictions were derived from the World Population Prospects 2017 Revision, classified by country, age, sex, and year (up to 2044).

### Statistical analysis

Retrieved from the GBD database, this study was designed to analyze the burden of mental disorders in Asia. The burden assessments are presented as age-standardized rates and absolute numbers of metrics based on the causes with their 95% uncertainty intervals (UIs), as well as the trends and changes from 1990 to 2019. DALYs, calculated by summing YLDs and YLLs, represents the total health loss due to mortality and morbidity in a population. Implemented in R through the package NORDPRED, a log-linear age–period–cohort model which limits linear trend projection and levels off exponential growth was fitted to recent trends, to predict the numbers and age-standardized rates of DALYs from 1990 to 2040 by country, sex, and age.

Based on geographical proximity and epidemiological similarity, 49 countries and territories in Asia were classified into 5 geographic sub-regions by the United Nations, including East Asia, West Asia, Southeast Asia, South Asia, and Central Asia. Our study examined the geographical variations in the epidemiological characteristics of mental disorders among Asian sub-regions.

The statistical procedures were conducted using the R program (version 4.1.3, R core team). All the analyses were deemed significant when *P* value < 0.05.

## Results

### Mental disorders burden in Asia

**Fig. 1:**
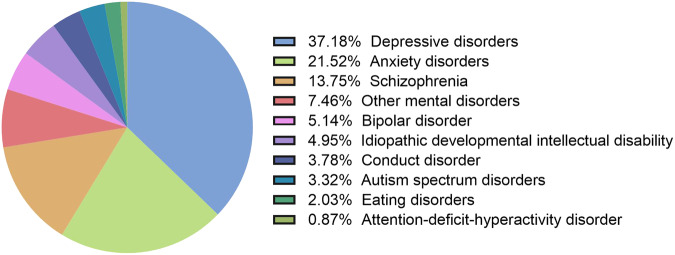
Distribution of DALYs due to mental disorders in Asia in 2019. DALYs, disability-adjusted life years.

Ranking as the eighth leading cause of disease burden, mental disorders contributed to 5.0% (4.0–6.1%) of total DALYs in Asia in 2019. As shown in Fig. 1, Depressive disorders (37.1%), followed by anxiety disorders (21.5%) and schizophrenia (13.8%), were the top three major components to mental disorder DALYs among the mental disorders analyzed in Asia. The top three mental disorders with the highest age-standardized rates of prevalence were anxiety disorders [3258.72 (95% UI: 2764.46–3805.07) per 100,000 population], depressive disorders [3196.16 (95% UI: 2881.31–3526.36) per 100,000 population] and Idiopathic developmental intellectual disability [1933.18 (95% UI: 1252.04–2626.36) per 100,000 population] (Table 1).

**Table 1:** Numbers and age-standardized rates of prevalence and disability-adjusted life-years due to mental disorders in Asian from 1990 to 2019.

	Age-standardized prevalence rate per 100,000 people (95% UI)		Prevalence, in millions (95% UI)		Age-standardized DALYs rate per 100,000 people (95% UI)		DALYs, in millions (95% UI)	
	1990	2019	1990	2019	1990	2019	1990	2019
Mental disorders								
Both	12191.63 (11176.78–13203.45)	11810.8 (10907.04–12753.33)	377.95 (344.08–411.41)	555.44 (512.92–598.47)	1452.2 (1080.16–1888.53)	1434.82 (1065.02–1867.27)	43.87 (32.47–57.18)	69.01 (51.04–89.71)
Female	12536.98 (11488.25–13518.06)	12118.42 (11164.55–13062.67)	187.69 (170.94–203.41)	283.33 (262.08–304.88)	1566.86 (1163.2–2044.69)	1530.4 (1135.87–1998.69)	23.04 (16.96–30.14)	36.52 (27.16–47.62)
Male	11842.47 (10822.14–12917.8)	11478.02 (10538.21–12477.08)	190.25 (172.43–208.42)	272.11 (250.07–295.62)	1340.69 (998.21–1743.46)	1339.29 (993.06–1751.91)	20.83 (15.38–27.22)	32.5 (24.1–42.41)
Depressive disorders								
Both	3267.56 (2947.53–3616.64)	3196.16 (2881.31–3526.36)	92.56 (82.68–103.25)	157.75 (141.95–174.58)	537.76 (377.81–732.69)	521.16 (366.84–710.41)	15.48 (10.8–21.4)	25.66 (18.04–34.98)
Female	4006.19 (3622.08–4440.94)	3842.63 (3458.28–4239.31)	55.94 (50.08–62.39)	94.38 (84.8–104.22)	663.28 (465.33–904.22)	626.96 (440.77–855.92)	9.41 (6.58–12.99)	15.34 (10.8–20.96)
Male	2554.3 (2300.28–2825.8)	2557.51 (2305.19–2824.76)	36.62 (32.61–40.93)	63.37 (56.99–70.39)	416.76 (291.15–568.56)	416.86 (292.42–568.39)	6.07 (4.23–8.35)	10.31 (7.22–14.08)
Anxiety disorders								
Both	3354.64 (2831.81–3924.82)	3258.72 (2764.46–3805.07)	102.12 (86.17–121.36)	155.77 (131.54–182.3)	319.61 (222.43–435.53)	311.17 (217.08–425.05)	9.83 (6.79–13.51)	14.85 (10.38–20.28)
Female	4139.3 (3501.43–4855.79)	4011.73 (3393.15–4699.24)	61.76 (51.9–73.66)	94.62 (79.8–111.04)	392.13 (273.93–535.21)	381.06 (264.46–520.25)	5.9 (4.08–8.11)	8.96 (6.25–12.29)
Male	2594.28 (2193.26–3027.63)	2525.05 (2140.65–2954.27)	40.36 (33.81–47.47)	61.15 (51.6–72)	249.54 (173.88–343.26)	243.31 (170.53–334.05)	3.92 (2.71–5.4)	5.89 (4.14–8.09)
Schizophrenia								
Both	290.41 (251.94–332.01)	293.03 (253.36–336.31)	8.39 (7.24–9.67)	14.74 (12.74–16.93)	186.29 (136.51–236.06)	188.69 (138.39–239.36)	5.42 (4–6.89)	9.49 (6.92–12.02)
Female	270.62 (234.51–309.31)	272.36 (235.35–312.72)	3.81 (3.28–4.39)	6.77 (5.84–7.78)	171.25 (125.15–216.21)	173.26 (126.06–218.81)	2.43 (1.79–3.08)	4.3 (3.12–5.44)
Male	309.06 (268.85–353.47)	313.02 (270.34–360.02)	4.58 (3.95–5.28)	7.97 (6.88–9.15)	200.48 (146.78–254.46)	203.62 (148.91–258.24)	2.99 (2.21–3.81)	5.19 (3.8–6.58)
Bipolar disorder								
Both	323.44 (270.75–380.01)	336.51 (279.5–397.79)	9.59 (7.92–11.38)	16.48 (13.73–19.51)	69.37 (42.59–106.52)	72.54 (44.24–111.57)	2.08 (1.26–3.2)	3.55 (2.16–5.47)
Female	327.96 (274.47–384.66)	337.46 (280.77–398.54)	4.76 (3.93–5.64)	8.16 (6.81–9.65)	69.68 (42.87–106.38)	72.08 (44.26–110.33)	1.02 (0.62–1.56)	1.74 (1.07–2.67)
Male	319.37 (267.65–375.25)	335.73 (279.72–397.26)	4.83 (3.99–5.74)	8.31 (6.94–9.86)	69.12 (42.36–105.99)	73.02 (44.49–112.73)	1.06 (0.64–1.63)	1.81 (1.11–2.79)
Other mental disorders								
Both	1397.04 (1071.27–1766.73)	1395.09 (1070.16–1764.89)	37.6 (28.9–47.98)	69.58 (53.22–87.97)	102.93 (66.25–156.64)	103.13 (66.29–156.76)	2.8 (1.79–4.27)	5.15 (3.31–7.85)
Female	1157.14 (887.04–1463.57)	1157.76 (887.42–1461.76)	15.18 (11.57–19.37)	28.83 (22.01–36.45)	84.23 (53.54–126.58)	84.66 (53.8–127.22)	1.11 (0.71–1.7)	2.11 (1.34–3.17)
Male	1631.11 (1248.52–2057.15)	1630.95 (1248.05–2058.39)	22.42 (17.22–28.53)	40.75 (31.08–51.57)	121.13 (78.74–183.37)	121.44 (78.66–183.79)	1.68 (1.08–2.55)	3.04 (1.97–4.6)
Conduct disorder								
Both	506.18 (361.8–663.17)	534.49 (385.05–698.85)	18.98 (13.58–24.87)	21.42 (15.36–28.06)	61.43 (34.51–97.28)	65.03 (36.45–102.16)	2.3 (1.3–3.63)	2.61 (1.47–4.09)
Female	339.96 (225.95–472.91)	368.17 (242.69–509.04)	6.17 (4.1–8.57)	7.05 (4.64–9.74)	41.07 (21.9–67.81)	44.59 (23.89–73.77)	0.75 (0.4–1.23)	0.85 (0.46–1.41)
Male	662.67 (489.46–856.66)	687.01 (509.2–879.17)	12.81 (9.49–16.57)	14.37 (10.65–18.39)	80.6 (46.14–126.06)	83.77 (48.28–131.14)	1.56 (0.89–2.43)	1.75 (1.01–2.74)
Autism spectrum disorders								
Both	340.78 (281.33–409.09)	334.05 (275.52–400.32)	11.18 (9.23–13.39)	15.02 (12.39–18.02)	51.92 (33.9–75.68)	51.02 (33.38–74.51)	1.71 (1.12–2.5)	2.29 (1.5–3.34)
Female	159.3 (129.06–193.94)	161.03 (130.09–195.88)	2.55 (2.06–3.1)	3.53 (2.85–4.3)	24.14 (15.9–35.17)	24.49 (16.03–35.78)	0.39 (0.26–0.57)	0.53 (0.35–0.78)
Male	514.72 (426.49–615.06)	500.21 (415.48–598.04)	8.63 (7.14–10.32)	11.49 (9.55–13.74)	78.49 (51.33–113.36)	76.45 (49.95–111.16)	1.32 (0.86–1.91)	1.75 (1.15–2.55)
Eating disorders								
Both	98.66 (73.88–127.25)	138.73 (103.36–178.34)	3.44 (2.57–4.43)	6.54 (4.84–8.4)	21.22 (13.17–31.43)	29.8 (18.56–44)	0.74 (0.46–1.1)	1.4 (0.87–2.06)
Female	124.32 (94.36–157.81)	171.63 (129.94–216.73)	2.11 (1.6–2.66)	3.94 (2.97–4.97)	26.53 (16.42–39.22)	36.72 (22.92–54.03)	0.45 (0.28–0.67)	0.84 (0.53–1.24)
Male	74.29 (52.35–98.07)	107.53 (76.25–142.81)	1.33 (0.93–1.75)	2.6 (1.83–3.48)	16.18 (9.78–24.61)	23.25 (13.99–35.3)	0.29 (0.17–0.44)	0.56 (0.34–0.86)
Idiopathic developmental intellectual disability								
Both	2144.47 (1398.14–2915.98)	1933.18 (1252.04–2626.36)	73.33 (48.1–99.52)	84.4 (54.55–114.86)	86.39 (47–139.21)	78.47 (42.76–127.11)	2.96 (1.62–4.78)	3.42 (1.86–5.54)
Female	2153.69 (1440.7–2892.14)	1955.53 (1292–2636.17)	35.73 (23.95–47.91)	41.53 (27.38–56.1)	86.2 (48.05–137.79)	78.99 (44.15–127.41)	1.44 (0.8–2.3)	1.67 (0.93–2.7)
Male	2135.76 (1367.95–2930.12)	1911.58 (1224.91–2618.64)	37.6 (24.19–51.45)	42.87 (27.37–58.82)	86.59 (46.22–140.27)	77.97 (41.72–126.86)	1.53 (0.82–2.47)	1.75 (0.93–2.84)
Attention-deficit/hyperactivity disorder								
Both	1255.53 (920.24–1673.21)	1132.8 (835.82–1492.15)	44.98 (32.81–60.42)	49.09 (36.29–64.38)	15.28 (8.72–25.56)	13.81 (7.9–23.34)	0.55 (0.31–0.93)	0.6 (0.34–1)
Female	690.98 (495.39–910.74)	625 (453.78–836.28)	12 (8.57–15.89)	13.18 (9.61–17.54)	8.37 (4.65–14.18)	7.59 (4.31–12.79)	0.15 (0.08–0.25)	0.16 (0.09–0.27)
Male	1788.96 (1312.62–2385.6)	1606.62 (1187.52–2110.68)	32.98 (24.18–44.39)	35.92 (26.56–46.8)	21.81 (12.41–36.59)	19.61 (11.24–33.14)	0.4 (0.23–0.68)	0.44 (0.25–0.73)

**Fig. 2:**
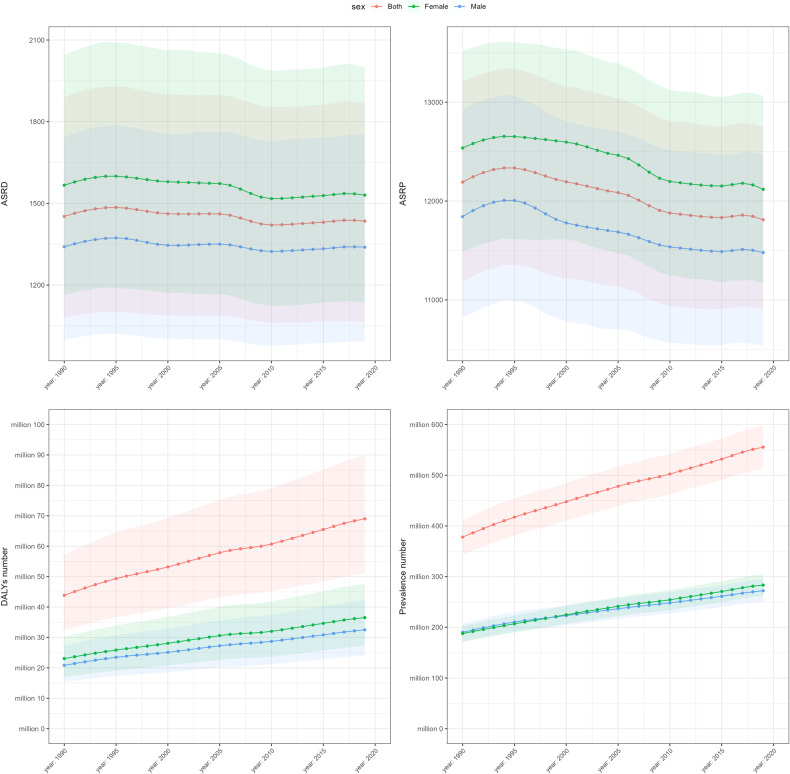
Age-standardized rates and numbers of DALYs and prevalence due to mental disorders in Asia for females, males and both sexes combined. DALYs disability-adjusted life years.

While the age-standardized rates of DALYs in mental disorders remained relatively stable over time, there was an increase among the ranking of these disorders (Table 2). Among all level 3 causes in GBD 2019, depressive disorders were ranked 22nd in 1990 and 15th in 2019, both within the top 25. There was also an upward trend in the estimated cases of mental disorders which increased from 377.9 million (95% UI: 344.1–411.4) in 1990 to 555.4 million cases (95% UI: 512.9–598.5) in 2019, with no marked increases identified in the age-standardized rates of prevalence of mental disorders and its subtypes between 1990 and 2019 (Table 1). As shown in Fig. 2, there was an increase in the absolute metric numbers of DALYs and prevalence in Asia by 47.0% and 57.5%, respectively. The increasing absolute numbers of DALYs and prevalence in Asia may imply the growing burden of mental disorders in Asian populations, with no marked increases in the age-standardized rates indicating that this trend was mainly influenced by demographic changes.

**Table 2:** Ranking of mental disorders among all level 3 GBD causes for age-standardized rates of DALYs, 1990–2019.

	1990 rank	2019 rank
Depressive disorders	22	15
Anxiety disorders	39	26
Schizophrenia	54	38
Bipolar disorder	88	80
Other mental disorders	74	59
Conduct disorder	97	82
Autism spectrum disorders	103	90
Eating disorders	132	117
Idiopathic developmental intellectual disability	83	73
Attention-deficit/hyperactivity disorder	142	134

### Mental disorders burden by sex and age

**Fig. 3:**
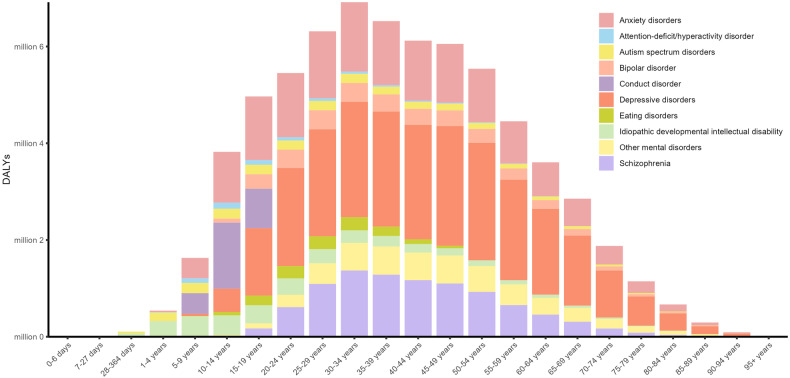
Numbers of DALYs due to mental disorders in Asia for both sexes combined. DALYs, disability-adjusted life years.

**Fig. 4:**
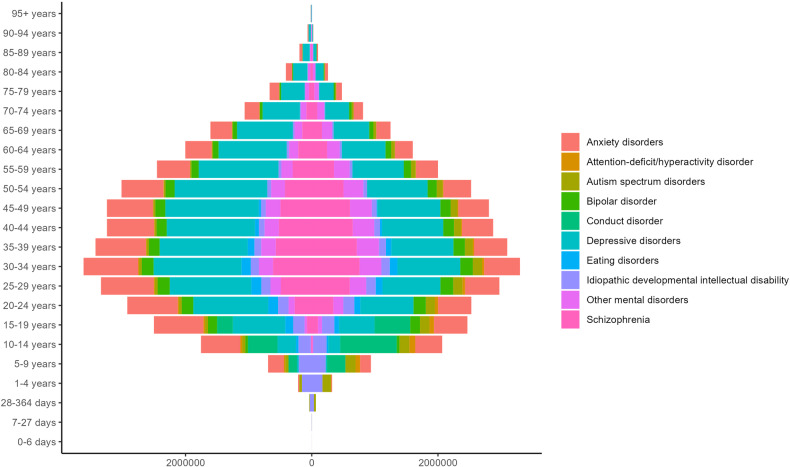
Numbers of DALYs due to mental disorders in Asia for females (left) and males (right). DALYs disability-adjusted life years.

**Fig. 5:**
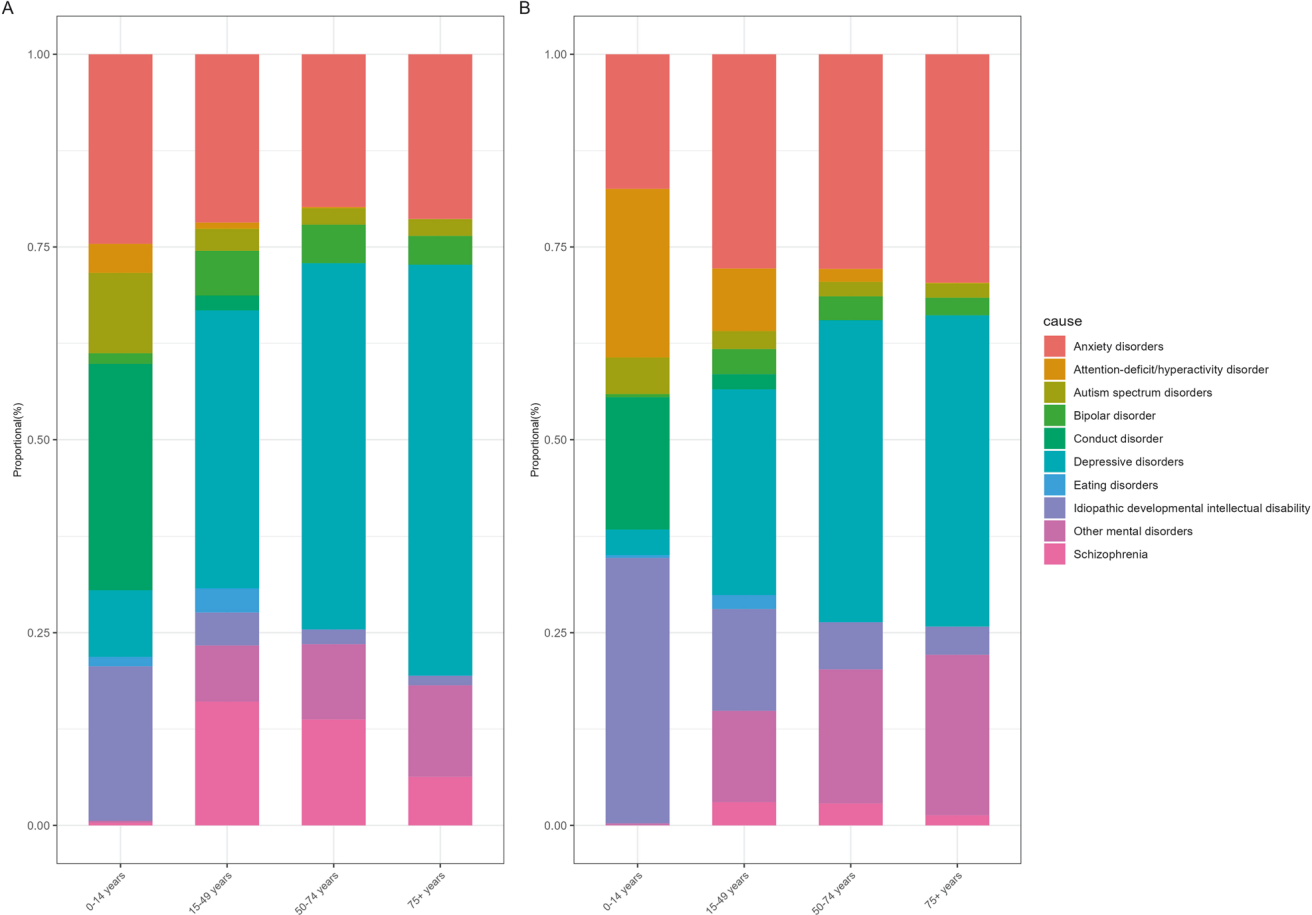
Distribution of mental disorders categories by age groups. Rates of DALYs (**A**) and prevalence (**B**) due to mental disorders in Asia in different age groups. DALYs disability-adjusted life years.

Different mental disorders had different age and sex distributions. For females, males and both sexes combined, the number of DALYs exhibited a consistent upward trend in childhood and adolescence, peaked at age 30-34 years, and steadily decreased after age 35 years. Autism spectrum disorders and idiopathic developmental intellectual disability emerged in individuals under the age of 5, with the burden of depressive disorders and anxiety disorders persisted and remained noticeable at older ages. The burden of anxiety disorders and depressive disorders rose abruptly during adolescence for both genders, which peaked at 10–14 years and 45–49 years, respectively (Figs. 3 and 4). As shown in Fig. 5, conduct disorder, anxiety disorders and idiopathic developmental intellectual disability constituted the primary contributors to mental disorder DALYs and prevalence among individuals below 15 years of age, whereas depressive disorders and anxiety disorders were the predominant components for individuals above the age of 15 years.

The age-standardized rates of prevalence [12118.42 (95% UI: 11164.55–13062.67) in females vs. 11478.02 (95% UI: 10538.21–12477.08) in males per 100,000 population] and DALYs [1530.4 (95% UI: 1135.87–1998.69) in females vs. 1339.29 (95% UI: 993.06–1751.91) in males per 100,000 population] in mental disorders for females were both higher than males (Table 1). The numbers of DALYs in mental disorders for females were also higher than males across all age groups, except for those below 15 years of age. Depressive disorders, eating disorders, and anxiety disorders were more common in females, compared with males, while conduct disorders, autism spectrum disorders, and attention-deficit hyperactivity disorder were more common in males (Table 1 and Fig. 4).

### Mental disorders burden by country

**Fig. 6:**
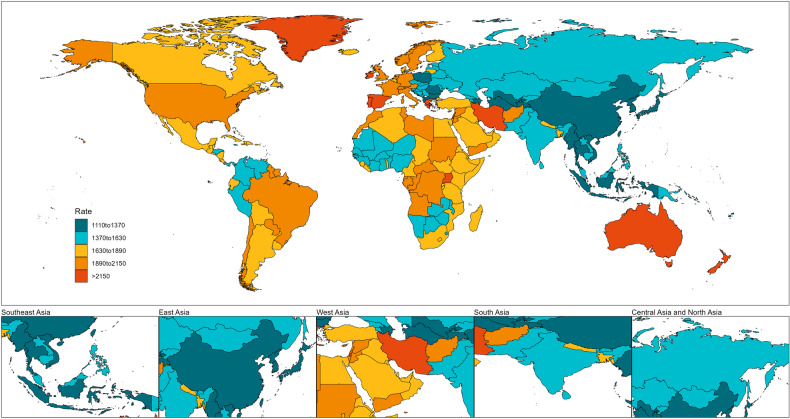
Age-standardized DALY rates in 2019 of mental disorders in 204 countries or territories. DALYs disability-adjusted life years.

As shown in Fig. 6 and Supplementary Table 1, the burden of mental disorders was significant in West Asia, while the top six highest age-standardized DALY rates of mental disorders were detected in Palestine [2396.89 (95% UI: 1749.94–3172.15) per 100,000 population], Iran [2295.81 (95% UI: 1702.21–3033.61) per 100,000 population], Lebanon [2126 (95% UI: 1552.27–2804.67) per 100,000 population], Afghanistan [2042.94 (95% UI: 1492.75–2692.05) per 100,000 population], Yemen [2041.02 (95% UI: 1499.65–2658.83) per 100,000 population], Bahrain[1980.82 (95% UI: 1449.68–2613.3) per 100,000 population]. The lowest age-standardized DALY rates were observed in Viet Nam, followed by Myanmar and Indonesia. The countries with the highest age-standardized DALY rates for depressive disorders were Palestine, Yemen, and Iran. Meanwhile. For anxiety disorders, Iran had the highest rates, followed by Cyprus and Lebanon.

**Fig. 7:**
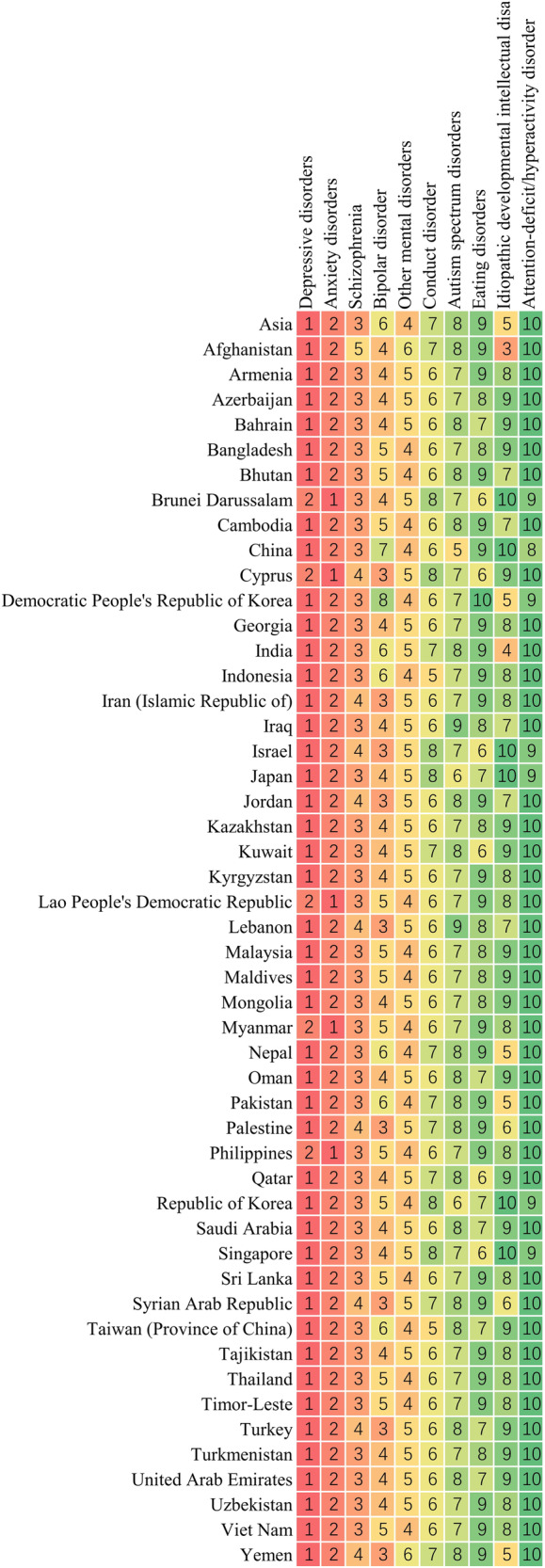
Ranking of age-standardized DALY rates for all mental disorders by Asian countries or territories, 2019. DALYs disability-adjusted life years.

**Fig. 8:**
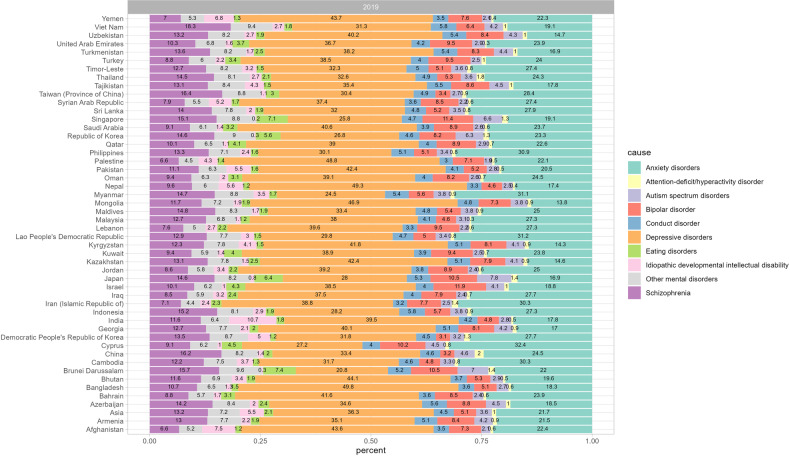
Distribution of age-standardized DALY rates in 2019 of mental disorders by Asian countries or territories. DALYs disability-adjusted life years.

As shown in Fig. 7, depressive disorders ranked as the primary cause of mental disorders in terms of the age-standardized DALY rates in 43 out of the 49 Asian countries and territories, while anxiety disorders were the leading cause in Brunei Darussalam, Cyprus, Lao People’s Democratic Republic, Myanmar, and Philippines. Idiopathic developmental intellectual disability is ranked relatively low, whereas in Afghanistan it holds the third position. Depressive disorders were the major components to mental disorder DALYs in Asia, especially in Bangladesh (49.8%) and Palestine (48.8%; Fig. 8).

### Prediction of mental disorders burden

**Fig. 9:**
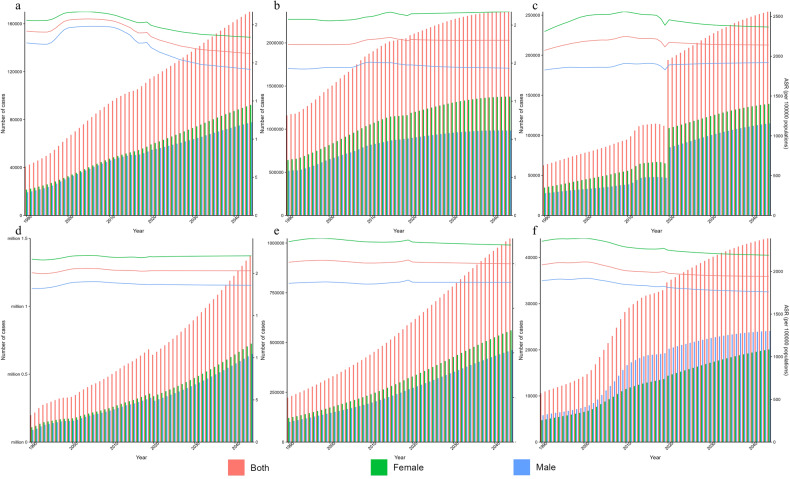
Trends in mental disorders over time by gender. Observed and predicted age-standardized rates and numbers of DALYs due to mental disorders in Palestine (**a**), Iran (Islamic Republic of) (**b**), Lebanon (**c**), Afghanistan (**d**), Yemen (**e**), and Bahrain (**f**), for females, males and both sexes combined. DALYs disability-adjusted life years.

**Fig. 10:**
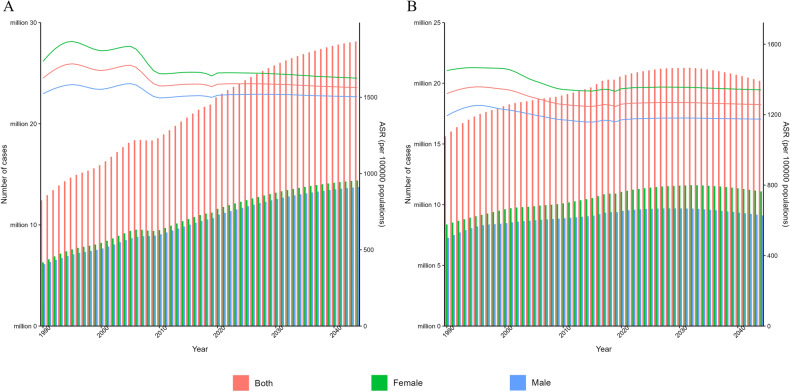
Trends in mental disorders over time by gender. Observed and predicted age-standardized rates and numbers of DALYs due to mental disorders in China (**A**) and India (**B**) for females, males and both sexes combined. DALYs disability-adjusted life years.

While the age-standardized DALY rates among the 8 elected countries, namely Palestine, Iran, Lebanon, Afghanistan, Yemen, Bahrain, China, and India, remained relatively stable, an increasing trend was observed in the number of DALYs, historically. The overall trend of stable rates and increasing numbers are predicted to continue for the future 25 years, with the exception of Palestine, where rates predicted to decline in the next 25 years. During the observed and predicted years, both rates and numbers in females were higher than in males, showing a similar trend of change. (Figs. 9 and 10).

### Relationship between mental disorders burden and SDI levels

**Fig. 11:**
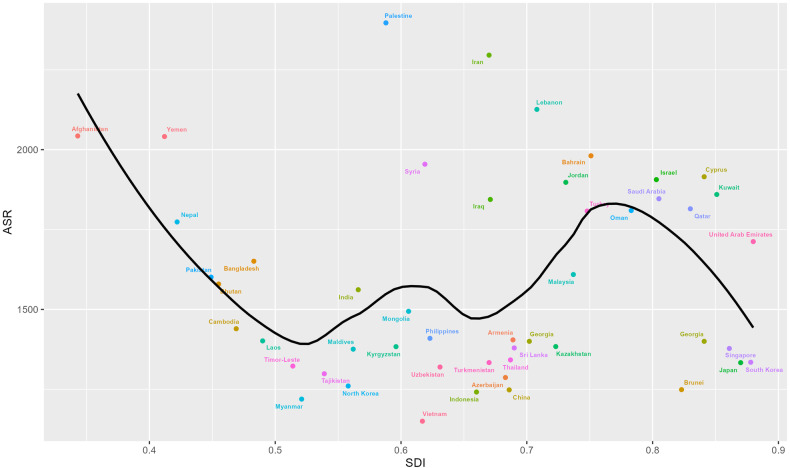
Age-standardized rates of DALYs due to mental disorders for Asia countries and territories by Socio-demographic Index, 1990–2019.

The observed national age-standardized DALY rates regarding SDI, versus the expected levels for each country based on SDI, were presented in Fig. 11. It turned out that there was no significant association between burden estimates of mental disorders and SDI levels for each country in Asia during the observation period. (Fig. 11).

## Discussion

Over the past 3 decades, we observed an ascending trend in the burden of mental disorders in Asia. Mental disorders ranked as the 13th highest contributor to DALYs among all level 2 causes in 1990, while in 2019, they advanced to the 8th position. Among all level 3 causes, depressive disorders and anxiety disorders were the primary contributors to the burden, ranking as 15th and 26th leading causes of DALYs, respectively.

Although the age-standardized prevalence and DALY rates in Asia remained relatively stable during the past 30 years, the numbers of prevalence and DALYs observed a rise of 47.0% and 57.5%, respectively, indicating that demographic changes may be the primary driving force behind this trend. Population growth, as well as aging, may mainly attribute to and continue to sustain this growth, highlighting the need for healthcare systems to the necessary treatment and care for this expanding demographic. However, obstacles to improving mental health persist, including discrimination, stigma, resource scarcity, unequal distribution of available resources, deficient public health policies, and lack of skills in primary health care, resulting in a range of social and economic burdens on individuals, families, employers, and society, spanning from the expenses associated with treatment to the losses in productivity [10, 11]. Given that, The commitment of governments and the international health community to allocate sufficient funding is crucial for the implementation of proven treatment and prevention programs.

The burden of mental disorders was greater in females compared to male population, while the burden concerning conduct disorders, autism spectrum disorders, and attention-deficit hyperactivity disorder was greater in males than females. As a result of globalization and urbanization, women may experience significant pressure, making them more susceptible to developing mental disorders. Chandra’s study showed that economic hardships, discrimination, stigma, gynecological problems, limited access to resources, low support from one’s family and marital disharmony were important contributors for the outset and chronicity of mental disorders among females [12]. Another study conducted by Silva, B. P. D. showed a high prevalence of perinatal mental disorders [13]. In the cultural context characterized by a preference for male offspring over female offspring, the gender of the infant was a significant predictor of postpartum depression [12]. Since the existence of mental disorders is indirectly associated with poor pregnancy outcomes, it is necessary for governments to develop preventive strategies, and mental health care delivery should be gender- and culture-sensitive [14]. According to the World Bank, more than 500 million women joined the global workforce, and female labor force participation rates in the East Asia and Pacific Region are relatively high. However, women are at a disadvantage in the labor market, with family-work conflict as well as racial and sexual harassment in the workplace significantly impacting their work [15, 16]. The depression of women would have an impact on the entire family, especially on children.

The burden of mental disorders rose abruptly during adolescence, peaked significantly in individuals of working age (16–65 years), and remains notable in people over the age of 65. Among children and adolescents, conduct disorder, anxiety disorders and idiopathic developmental intellectual disability constituted a substantial percentage of diseases. Mental diseases would impact not only their day-to-day function, but also their education, physical health and even life expectancy [17]. Without timely treatment or intervention, these mental health problems may result in self-harm and suicide, even continuing into adulthood [18]. While learning disabilities, chronic physical ill health, domestic, physical or sexual abuse, and parental psychopathology appear to be significant risk factors, parental support, good peer relationships, and self-esteem seem to be protective factors [19]. In addition, studies indicated that elevated stress levels during pregnancy can negatively impact the structure and function of the fetus’s brain, potentially resulting in subsequent mood and anxiety disorders.

In adults, depressive disorders and anxiety disorders were the primary contributors. Although only eating disorders were recognized as the underlying causes of mortality, the relationship between mental disorders and death is very complex. Studies indicated that patients with mental disorders tend to face premature mortality and lost more years of life, compared with individuals with non-major mental disorders, primarily attributable to factors like infections, chronic diseases, suicide, and other causes [2, 20]. Because the GBD database commonly attributes deaths to the ultimate cause of mortality, the increased mortality that impacts patients with mental disorders (especially schizophrenia, depressive disorders, and bipolar disorder) was underestimated [6]. The study conducted by Lambert revealed that individuals with severe mental illness, including schizophrenia, bipolar disorder and major depressive disorder, faced a heightened risk for cardiovascular disease (CVD) as well as CVD-related death [21]. In most Asian countries, the burden of the three severe mental illnesses mentioned above ranked high. In addition to CVD, respiratory disease was also attributed to this excess mortality, with asthma, pneumonia, and chronic obstructive pulmonary disease being more prevalent among individuals with schizophrenia than in the general population [22]. Furthermore, adverse health behaviors among them like smoking, substance use and poor diet would exacerbate the course of chronic medical conditions [2, 20].

The substantial burden of mental disorders and a scarcity of resources allocated for mental health in nearly all Asian countries poses presents formidable challenges for Asia [23]. According to WHO Mental Health Atlas 2020, the number of psychiatrists in almost all countries and territories in Asia-Pacific was below the Organization for Economic Cooperation and Development average of 18.1 per 100,000 population, and there were, on average, fewer than 5 per 100,000 population mental health nurses in low- and middle-income Asia-Pacific countries and territories. Given that, mental health care workforce should be appropriately supplied to guarantee access. Although there is no notable association between the burden of mental disorders and SDI, limited mental healthcare resources in low- and middle-income countries would result in a potential treatment gap [24]. Furthermore, in the latter half of the 20th century, the aging of Asia’s population progressed rapidly in the less developed countries, representing significant challenges related to caring for and supporting the elderly and their families [25]. The stigma linked to mental health would constitute another obstacle, leading to decreased accessibility to healthcare services, delayed help seeking, subpar treatment, and poor outcomes. Inclusive and comprehensive mental health legislations and policies are needed for addressing stigma requires [26].

Mental health and mental illness have been redefined to be viewed as a continuum from health to illness, highlighting the significance of interventions of promotion and prevention [5, 27]. To cope with the mental health requirements among diverse and geographically scattered populations in Asia, an emerging interdisciplinary domain called mobile health offers a novel approach that could be used to improve individuals’ mental health literacy, reduce the impact of stigmatization, enhance community outreach and engagement, and assist illness self-management [23]. As a resource for mental health care and referral, primary care physicians, compared with psychiatrists, have potential advantages including easier access for patient, firsthand information of patients’ backgrounds, and provision of continuing care. Through trainings and policy development, a large group of primary care physicians may help alleviate the shortage of mental health care workforce [28]. To deal with the anticipated rise in the burden of mental disorders, it is necessary to expand the implementation of effective prevention and treatment projects.

This study investigated the prevalence and DALYs over the past 30 years in Asian countries, as well as ranks of the burden of mental disorders and their correlations with SDI. We also made predictions for the burden in 8 selected countries over the next 25 years. However, our study also come with some limitations. First, the use of ICD and DSM diagnostic criteria, which were mainly established in developed countries, may not be sensitive across all cultures [29]. Second, there was some bias among population prediction in Lebanon. Third, the deaths related to mental disorders have been underestimated, because, besides eating disorders, other factors such as suicide and ischemic heart disease related to depressive disorders could also contribute to the mortality of patients with mental disorders [1].

## In conclusion

all mental disorders ranked higher in 2019, compared in 1990 in Asia. To reduce this burden, urgent measures for prevention, treatment and rehabilitation for mental disorders need to be taken by Asian governments.

### Supplementary information

Supplementary Table 1

## Data Availability

The data used in these analyses are available on the Global Health Data Exchange GBD 2019 website, and the codes used for analysis and projections can be obtained from the corresponding author upon reasonable request.
