# Supplementary material for: The burden of mental disorders in Asian countries, 1990–2019: an analysis for the global burden of disease study 2019
Source: Transl Psychiatry. 2024 Mar 28;14:167. doi: 10.1038/s41398-024-02864-5 (PMC10978857; doi:10.1038/s41398-024-02864-5)
Supplement: Supplementary file 1 — Supplementary Table 1 [file 41398_2024_2864_MOESM1_ESM.docx]

**Supplementary Table 1. Age-standardized rates of disability-adjusted life-years due to mental disorders in 2019 in Asian countries and territories.**

|  | Mental disorders | Autism spectrum disorders | Attention-deficit/hyperactivity disorder | Conduct disorder | Idiopathic developmental intellectual disability | Other mental disorders | Schizophrenia | Depressive disorders | Bipolar disorder | Anxiety disorders | Eating disorders |
| --- | --- | --- | --- | --- | --- | --- | --- | --- | --- | --- | --- |
| Asia | 1434.82(1065.02-1867.27) | 51.02(33.38-74.51) | 13.81(7.9-23.34) | 65.03(36.45-102.16) | 78.47(42.76-127.11) | 103.13(66.29-156.76) | 188.69(138.39-239.36) | 521.16(366.84-710.41) | 72.54(44.24-111.57) | 311.17(217.08-425.05) | 29.8(18.56-44) |
| Afghanistan | 2042.94(1492.75-2692.05) | 43.4(28.57-62.84) | 12.02(6.58-20.18) | 71.23(40.54-111.27) | 154.19(85.55-245.89) | 106.1(67.83-159.2) | 135.64(96.4-180.78) | 890.19(608.04-1238.01) | 148.22(87.27-232.22) | 457.25(305.66-639.08) | 24.71(15.19-36.82) |
| Armenia | 1404.67(1033.78-1855.4) | 58.73(38.19-85.04) | 12.99(7.39-22.2) | 72.21(40.41-114.33) | 30.34(12.92-53.21) | 107.94(68.26-164.15) | 182.8(128.03-242.67) | 492.75(342.64-679.56) | 117.56(68.99-183.15) | 302.23(201.47-426.98) | 27.13(16.73-40.08) |
| Azerbaijan | 1286.83(946.73-1694.65) | 58.17(37.71-83.25) | 13.01(7.15-22.24) | 72.06(40.52-114) | 25.16(10.5-43.79) | 108.46(69.07-164.79) | 182.7(126.25-241.41) | 445.11(306.49-621.39) | 113.53(67.74-177.05) | 237.74(158.55-333.45) | 30.91(18.77-47.15) |
| Bahrain | 1980.82(1449.68-2613.3) | 48.28(31.69-70.79) | 12.5(6.88-21.21) | 71.83(40.58-112.55) | 33.62(15.15-58.5) | 112.97(72.48-171.63) | 173.45(120.67-232.31) | 823.25(563.09-1149.98) | 168.85(99.25-260.03) | 474.19(313.11-664.87) | 61.88(38.78-93.06) |
| Bangladesh | 1650.82(1208.91-2188.29) | 44.62(29.19-66.48) | 9.64(5.45-16.58) | 58.68(31.45-93.58) | 20.64(6.75-39.1) | 107.91(68.77-163.82) | 176.63(122.29-234.48) | 822.2(563-1140.18) | 83.66(49.11-130.34) | 302.17(200.09-428.04) | 24.67(15.06-37.25) |
| Bhutan | 1579.42(1167.94-2076.68) | 45.4(29.69-65.39) | 7.85(4.34-13.46) | 58.4(32.2-93.6) | 53.58(26.56-89.8) | 109.21(69.06-165.68) | 183.3(126.71-244.46) | 696.86(482.55-963.04) | 84.3(49.81-129.82) | 309.87(207.65-438.49) | 30.64(18.69-45.77) |
| Brunei Darussalam | 1248.85(911.1-1653.67) | 87.06(57.08-125.4) | 17.95(9.89-31.35) | 64.9(35.95-104.31) | 4.22(0.46-10.48) | 119.89(75.96-179.05) | 195.93(134.14-261.93) | 260.29(177.62-360.71) | 131.4(76.72-206.11) | 274.96(186.03-384.95) | 92.25(58-136.77) |
| Cambodia | 1439.48(1054.47-1897.56) | 46.93(30.09-67.88) | 11.18(6.23-19.1) | 66.06(37.04-106.33) | 52.71(26.13-90.02) | 107.41(68.18-162.72) | 175.94(123.94-234.11) | 456.36(314.5-643.72) | 68.49(40.65-107.21) | 436.25(289.9-620.04) | 18.15(11.02-27.35) |
| China | 1248.16(922.38-1627.36) | 57.34(37.43-83.77) | 25.34(14.67-42.92) | 56.93(31.6-91.72) | 16.95(6.39-30.1) | 102.03(65.25-155.43) | 202.42(151.35-252.8) | 416.95(292.97-574.39) | 39.64(24.37-60.83) | 306.02(214.72-419.04) | 24.54(14.98-36.33) |
| Cyprus | 1915.2(1390.06-2540.68) | 85.91(56.07-123.95) | 15.78(8.69-26.73) | 76.64(43.31-122.08) | 19.45(6.62-34.87) | 118.35(75.46-176.21) | 174.94(121.59-230.79) | 521.45(352.92-722.35) | 196.12(114.59-300.56) | 619.97(413.08-874.55) | 86.59(54.24-127.19) |
| Democratic People's Republic of Korea | 1260.33(931.94-1658.87) | 40.26(25.94-58.92) | 16.13(9.11-27.45) | 56.61(31.27-91.46) | 63.39(34.38-102.76) | 109.67(69.88-165.52) | 170.51(119.06-229.89) | 401.11(275.98-561.2) | 39.64(23.08-62.37) | 348.51(231.66-487.37) | 14.5(8.86-21.48) |
| Georgia | 1400.08(1033.63-1848.18) | 58.19(37.47-84.55) | 13(7.19-22.15) | 71.92(40.62-113.25) | 29.92(12.81-52.49) | 107.77(68.72-162.79) | 178.27(122.49-236.77) | 561.36(387.4-777.16) | 113.58(66.7-175.36) | 238.67(159.5-334.21) | 27.4(16.89-40.86) |
| India | 1561.63(1155.05-2027.44) | 43.71(28.6-63.91) | 7.03(3.92-12.22) | 66.03(36.93-103.56) | 167.55(95.27-268.3) | 99.55(64.24-152.52) | 180.39(130.01-229.69) | 616.13(433.49-834.61) | 75.25(46.58-114.78) | 278.5(196.1-378.45) | 27.5(17-40.62) |
| Indonesia | 1241.4(928.68-1631.94) | 47.47(30.87-69.11) | 11.57(6.58-19.95) | 72.22(40.94-113.43) | 35.76(17.34-61.14) | 100.77(65.18-152.97) | 189.27(140.34-241.16) | 350.26(242.89-488.45) | 71.25(43.86-108.56) | 338.9(239.58-465.15) | 23.92(14.82-35.38) |
| Iran (Islamic Republic of) | 2295.81(1702.21-3033.61) | 56.43(37.04-81.74) | 32.42(18.12-55.49) | 72.57(41.03-116.1) | 55.91(28.07-94.44) | 100.59(64.47-153.43) | 162.02(118.26-205.92) | 890.32(605.75-1247.77) | 175.84(107.91-270) | 695.76(483.28-954.7) | 53.94(33.53-80.2) |
| Iraq | 1844.17(1358.19-2442.18) | 43.77(28.67-64.01) | 12.63(7.06-21.37) | 74.63(42.32-116.45) | 58.2(28.68-99.54) | 107.98(68.4-162.74) | 156.52(109.93-208.94) | 690.87(476.11-965.21) | 145.3(85.89-225.11) | 510.11(337.29-730.94) | 44.17(26.58-66.36) |
| Israel | 1906.2(1394.84-2492.71) | 78.09(51.47-112.14) | 19.4(10.92-33.03) | 76.49(43.62-121.52) | 19.15(6.24-34.1) | 118.92(74.47-178.76) | 191.6(135.54-251.96) | 734.19(506.39-1009.93) | 227.08(133.85-349.41) | 359.07(241.31-505.33) | 82.2(51.41-123.49) |
| Japan | 1333.33(981.44-1749.87) | 103.78(67.7-149.66) | 18.22(10.2-30.91) | 71.29(40.64-112.29) | 10.93(2.5-21.62) | 109.1(70.05-164.6) | 195.19(140.85-247.93) | 373.85(260.7-510.79) | 140.44(86.05-214.53) | 224.86(158.47-308.42) | 85.68(55.06-125.99) |
| Jordan | 1897.72(1395.76-2501.56) | 45.97(30.11-67.85) | 12.3(6.71-20.6) | 72.09(40.83-113.08) | 63.58(31.45-106.14) | 110.34(70.39-166.35) | 164.01(113.86-221.07) | 743.53(505.02-1027.87) | 169.64(100.21-263.45) | 475.06(315.69-666.71) | 41.2(25.33-62.83) |
| Kazakhstan | 1383.69(1024.44-1822.97) | 56.7(36.9-83.07) | 12.86(7.19-21.84) | 71.24(39.76-113.11) | 21.02(8.06-37.1) | 107.33(68.63-162.61) | 181.17(125.2-243.01) | 586.6(410.93-815.96) | 109.63(64.29-168.69) | 202.17(134.28-287.98) | 34.97(21.4-52.88) |
| Kuwait | 1859.75(1366.45-2471.55) | 46.58(30.59-68.5) | 12.18(6.73-20.74) | 72(40.65-113.22) | 26.88(11.42-46.98) | 110.47(69.72-166.11) | 175.6(121.02-234.34) | 723.95(488.4-1001.48) | 175.31(101.07-273.07) | 442.74(294.31-632.13) | 74.05(46.07-110.1) |
| Kyrgyzstan | 1383.45(1014.55-1828.43) | 57.27(37.7-83.36) | 12.86(7.11-22.06) | 71.11(39.55-113.24) | 56.31(28.08-93.37) | 108.36(69.49-163.48) | 169.5(120.38-224.9) | 577.77(398.58-809.09) | 112.13(65.21-174.37) | 198(133.77-278.07) | 20.14(12.24-30.39) |
| Lao People's Democratic Republic | 1401.44(1030.57-1848.76) | 47.49(30.96-68.7) | 11.17(6.29-19.3) | 65.79(37.32-106.91) | 41.54(19.88-70.52) | 108.56(69.72-163.14) | 180.72(126.68-244.15) | 417.54(287.05-590.16) | 70.24(41.67-110.33) | 437.1(296.18-605.86) | 21.29(13.05-31.82) |
| Lebanon | 2126(1552.27-2804.67) | 46.19(30.17-67.87) | 12.1(6.78-20.56) | 70.49(40.63-110.49) | 56.78(28.45-94.67) | 106.91(68.23-160.7) | 161.44(111.11-213.39) | 842.01(576.15-1165.86) | 203.01(122.61-312.65) | 579.41(385.85-817.32) | 47.66(29.22-70.04) |
| Malaysia | 1609.21(1174.71-2136.23) | 49.62(32.35-72.88) | 5.26(2.94-9.4) | 66.2(37.15-106.4) | 16.99(5.63-31.11) | 109.23(69.32-165.27) | 204.76(146.03-275.74) | 611.66(417.41-853.92) | 73.32(43.02-115.49) | 439.81(295.29-622.65) | 32.37(20.19-47.75) |
| Maldives | 1375.63(1016.74-1804.8) | 52.72(34.4-77.12) | 11.75(6.55-19.94) | 66.64(37.5-105.83) | 23.49(9.56-41.87) | 113.7(71.89-171.47) | 203.8(142.83-276.02) | 459.45(317.74-648.23) | 73.71(42.38-116.61) | 344.09(230.12-487.4) | 26.27(16.01-39.14) |
| Mongolia | 1494.02(1100.9-1970.61) | 56.65(36.85-82.16) | 12.85(7.17-22.06) | 71.11(40.55-111.38) | 27.73(11.31-48.89) | 107.93(68.61-163.63) | 175.1(122.07-231.11) | 700.27(480.9-973.1) | 108.38(65.02-169.55) | 205.45(138.21-285.05) | 28.54(17.88-42.63) |
| Myanmar | 1219.29(894.72-1612.43) | 46.6(30.61-69.08) | 11.06(6.13-18.89) | 65.67(36.68-107.05) | 43.17(20.6-74.14) | 107.27(68.47-162.24) | 179.12(126.98-238.11) | 298.19(203.44-421.71) | 68.31(41.07-107.05) | 379.27(253.58-535.66) | 20.63(12.41-30.82) |
| Nepal | 1773.74(1309.23-2335.08) | 43.9(28.65-64.13) | 7.68(4.26-13.15) | 58.9(32-93.8) | 99.9(54.97-162.85) | 106.04(67.06-161.37) | 169.46(118.16-224.01) | 874.04(603.14-1205.18) | 82.26(48.93-126.33) | 309.51(205.76-434.63) | 22.05(13.63-33.46) |
| Oman | 1809.53(1319.09-2394.99) | 46.3(30.65-67.91) | 12.87(7.17-21.83) | 71.64(40.54-113.56) | 36.28(16.33-61.62) | 114.66(72.68-173.44) | 170.41(117.47-226.89) | 707.8(481.49-976.56) | 148.78(87.78-233.39) | 444.2(297.1-633.5) | 56.6(34.6-85.47) |
| Pakistan | 1601.02(1188.71-2105.84) | 45.31(29.81-66.38) | 7.99(4.41-13.83) | 65.76(36.97-103.59) | 88.79(46.08-147.7) | 100.42(64.83-152.14) | 177.39(128.09-223.74) | 678.27(474.27-937.43) | 82.96(51.89-126.33) | 328.23(231.49-452.55) | 25.9(15.97-38.38) |
| Palestine | 2396.89(1749.94-3172.15) | 44.41(28.86-64.37) | 12.06(6.8-20.44) | 71.62(40.94-112.19) | 103.39(57.12-165.87) | 107.59(68.35-162.59) | 157.26(109.22-209.7) | 1168.68(802.95-1624.31) | 169.32(99.15-260.87) | 529.11(349.29-742.03) | 33.45(20.39-50.84) |
| Philippines | 1409.45(1047.97-1857.04) | 48.23(31.59-70.12) | 11.59(6.58-19.94) | 72.21(41.59-114.17) | 34.16(14.87-60.37) | 100.68(64.29-154) | 187.8(138.44-238.72) | 424.69(292.61-587.44) | 72.5(44.64-110.26) | 435.03(307.6-593.27) | 22.56(14.06-33.41) |
| Qatar | 1814.89(1326.29-2387.05) | 52.57(34-75.94) | 13.53(7.5-23.2) | 73.32(41.73-115.43) | 20.17(7.46-36.24) | 118.5(75.23-177.7) | 182.5(125.67-245.02) | 708.38(487.12-981.53) | 161.18(93.38-249.25) | 410.74(275.19-576.98) | 74.02(46.09-112.38) |
| Republic of Korea | 1334.59(975.74-1758.55) | 84.14(53.82-121.94) | 16.77(9.35-28.79) | 61.34(33.04-98.35) | 4.09(0.57-9.71) | 120.48(75.81-181.46) | 194.88(134.03-259.99) | 357.64(248.35-491.66) | 109.01(64.81-170.97) | 310.93(205.17-439.93) | 75.32(47.19-111.8) |
| Saudi Arabia | 1846.33(1356.11-2433.4) | 47.36(30.76-68.71) | 11.32(6.33-19.28) | 71.35(40.55-111.27) | 26.66(11.33-47.09) | 111.73(70.53-167.37) | 167.78(117.19-225.18) | 748.71(515.68-1040.33) | 164.41(96.78-258.12) | 437.26(293.08-616.27) | 59.77(37.38-90.12) |
| Singapore | 1377.69(1005.25-1808.32) | 90.26(58.81-131.74) | 17.58(9.73-30.43) | 64.53(35.17-103.05) | 3.37(0.26-8.77) | 120.85(76.95-182.44) | 208.19(143.04-276.23) | 355.22(243.19-490.02) | 157.7(93.48-244.11) | 262.66(175.28-372.5) | 97.32(60.5-145.09) |
| Sri Lanka | 1379.43(1012.19-1823.9) | 48.96(32.2-71.67) | 11.1(6.21-19.12) | 65.96(36.78-105.28) | 28.25(12.28-49.12) | 107.19(69.36-162.02) | 193.52(136.08-258.44) | 440.87(301.85-614.43) | 72.31(41.92-111.11) | 385.41(257.33-549.06) | 25.84(15.94-39.35) |
| Syrian Arab Republic | 1954.39(1449.75-2579.27) | 43.17(28.56-63.41) | 11.7(6.37-20.01) | 71.11(39.2-112.14) | 100.92(53.67-164.61) | 106.6(67.93-162.51) | 154.36(107.5-206.15) | 731.25(493.6-1010.88) | 166.26(96.1-256.87) | 535.56(359.02-757.58) | 33.46(20.43-50.37) |
| Taiwan (Province of China) | 1246.89(913.48-1639.49) | 33.31(22.06-47.89) | 11.52(6.49-19.91) | 61.47(34.36-98.12) | 13.4(5.29-23.4) | 109.67(69.82-165.59) | 204.4(143.93-272.28) | 378.76(261-532.4) | 42.22(24.6-65.98) | 354.69(234.32-504.21) | 37.44(22.97-56.08) |
| Tajikistan | 1298.58(958.28-1707.94) | 57.82(38.29-83.4) | 12.91(7.19-21.83) | 71.22(40.35-113.69) | 55.9(27.09-94.62) | 108.79(69.03-164.01) | 170.56(119.32-226.7) | 459.08(317.05-636.89) | 112.12(65.75-173.47) | 230.99(152.87-321.24) | 19.2(11.74-28.83) |
| Thailand | 1341.92(984.48-1760.11) | 47.84(31-69.51) | 23.91(13.45-39.81) | 66.22(37.05-105.89) | 35.97(16.1-63.05) | 108.46(69.28-164.63) | 194.86(135.48-259.92) | 438.07(298.87-612.27) | 71.5(42.96-112.09) | 326.53(218.56-463.63) | 28.57(17.71-42.55) |
| Timor-Leste | 1322.69(969.05-1743.62) | 47.73(31.2-69.04) | 11.2(6.36-19.22) | 66.1(37.03-105.91) | 42.47(20.2-73.27) | 108.25(68.69-164.15) | 168.58(118.8-222.4) | 427.71(290.48-599.53) | 67.94(40.62-105.83) | 362.29(238.35-512.72) | 20.43(12.56-30.68) |
| Turkey | 1807.78(1317.36-2378.19) | 44.86(29.48-66.22) | 18.21(10.23-30.59) | 71.78(40.82-113.18) | 40.31(17.11-70.76) | 108.62(69.29-163.9) | 159.78(115.75-205.46) | 696.29(477.3-952.56) | 172.22(100.51-268.36) | 433.74(291.28-616.96) | 61.96(37.64-92.2) |
| Turkmenistan | 1333.59(982.66-1756.06) | 58.13(38.03-84.55) | 13.04(7.31-22.14) | 71.71(40.28-113.31) | 22.87(9.27-40.1) | 109.23(69-166.41) | 181.79(125-241.75) | 508.89(350.89-698.22) | 110.06(64.42-169.36) | 225.03(149.95-318.5) | 32.83(19.96-49.22) |
| United Arab Emirates | 1712.22(1256.73-2253.01) | 50.47(32.96-73.53) | 5.39(2.97-9.22) | 71.59(40.22-112.65) | 26.84(11.05-46.91) | 117.05(74-177.84) | 176.55(123.7-235.04) | 628.63(425.93-866.17) | 163.04(94.49-248.43) | 408.54(273.34-576.6) | 64.14(39.57-96.76) |
| Uzbekistan | 1319.62(969.87-1743.88) | 57.31(37.23-83.98) | 12.89(7.24-22.02) | 71.21(40.25-113.55) | 34.98(15.54-61.96) | 108.3(68.36-163.57) | 174.02(120.84-229.91) | 530.95(361.44-746.01) | 111(64.91-170.27) | 194.11(130.46-271.31) | 24.84(15.04-37.35) |
| Viet Nam | 1150.17(851.48-1513.05) | 48.37(31.56-71.32) | 11.25(6.35-19.1) | 66.51(37.75-105.21) | 30.56(13.93-53.79) | 108.69(68.88-164.51) | 210.69(147.39-280.96) | 360.23(244.42-502.95) | 73.05(42.49-114.06) | 220.24(149.42-312.99) | 20.59(12.13-30.8) |
| Yemen | 2041.02(1499.65-2658.83) | 43.48(28.3-62.77) | 8.86(4.92-15.28) | 71.49(40.35-110.88) | 138.17(76.43-224.4) | 107.76(69.33-163.77) | 143.33(100.57-191.25) | 890.99(607.6-1235.38) | 155.44(91.28-240.27) | 455.44(300.23-631.68) | 26.06(15.69-38.84) |
